# Assessment of metastatic lymph nodes in head and neck squamous cell carcinomas using simultaneous ^18^F-FDG-PET and MRI

**DOI:** 10.1038/s41598-020-77740-5

**Published:** 2020-11-27

**Authors:** Jenny Chen, Mari Hagiwara, Babak Givi, Brian Schmidt, Cheng Liu, Qi Chen, Jean Logan, Artem Mikheev, Henry Rusinek, Sungheon Gene Kim

**Affiliations:** 1 Department of Radiology, Center for Biomedical Imaging (CBI), Center for Advanced Imaging Innovation and Research (CAI2R), New York University School of Medicine, 660 First Avenue, New York, NY 10016 USA; 2 Department of Otolaryngology-Head and Neck Surgery, New York University School of Medicine, New York, NY USA; 3 Department of Oral and Maxillofacial Surgery, Bluestone Center for Clinical Research, New York University College of Dentistry, New York, NY USA; 4 Department of Pathology, New York University School of Medicine, New York, NY USA; 5 Department of Radiology, Weill Cornell Medical College, New York, NY USA

**Keywords:** Head and neck cancer, Image processing, Diagnostic markers

## Abstract

In this study, we investigate the feasibility of using dynamic contrast enhanced magnetic resonance imaging (DCE-MRI), diffusion weighted imaging (DWI), and dynamic positron emission tomography (PET) for detection of metastatic lymph nodes in head and neck squamous cell carcinoma (HNSCC) cases. Twenty HNSCC patients scheduled for lymph node dissection underwent DCE-MRI, dynamic PET, and DWI using a PET-MR scanner within one week prior to their planned surgery. During surgery, resected nodes were labeled to identify their nodal levels and sent for routine clinical pathology evaluation. Quantitative parameters of metastatic and normal nodes were calculated from DCE-MRI (v_e_, v_p_, PS, F_p_, K^trans^), DWI (ADC) and PET (K_i_, K_1_, k_2_, k_3_) to assess if an individual or a combination of parameters can classify normal and metastatic lymph nodes accurately. There were 38 normal and 11 metastatic nodes covered by all three imaging methods and confirmed by pathology. 34% of all normal nodes had volumes greater than or equal to the smallest metastatic node while 4 normal nodes had SUV > 4.5. Among the MRI parameters, the median v_p_, F_p_, PS, and K^trans^ values of the metastatic lymph nodes were significantly lower (*p* = <0.05) than those of normal nodes. v_e_ and ADC did not show any statistical significance. For the dynamic PET parameters, the metastatic nodes had significantly higher k_3_ (*p* value = 8.8 × 10^−8^) and K_i_ (*p* value = 5.3 × 10^−8^) than normal nodes. K_1_ and k_2_ did not show any statistically significant difference. K_i_ had the best separation with accuracy = 0.96 (sensitivity = 1, specificity = 0.95) using a cutoff of K_i_ = 5.3 × 10^−3^ mL/cm^3^/min, while k_3_ and volume had accuracy of 0.94 (sensitivity = 0.82, specificity = 0.97) and 0.90 (sensitivity = 0.64, specificity = 0.97) respectively. 100% accuracy can be achieved using a multivariate logistic regression model of MRI parameters after thresholding the data with K_i_ < 5.3 × 10^−3^ mL/cm^3^/min. The results of this preliminary study suggest that quantitative MRI may provide additional value in distinguishing metastatic nodes, particularly among small nodes, when used together with FDG-PET.

## Introduction

Head and neck cancer (HNC) represents approximately 4% of invasive cancers diagnosed annually in the United States^1^. Approximately 65,000 Americans develop HNC each year and nearly 14,500 patients with HNC die from it each year. Worldwide, more than 500,000 individuals will develop HNC each year, ranking it as the sixth most common cancer. These cancers predominately originate from mutations in the squamous cells and approximately two thirds of patients will present with locally advanced disease with either large disease at the primary site and/or spread to regional lymph nodes. Approximately 50% of patients presenting with these advanced diseases survive for more than 5 years.

Accurate identification and characterization of lymph node metastasis by non-invasive imaging has important therapeutic and prognostic significance in patients with newly diagnosed HNC as well as in evaluating treatment response^2–5^. It is crucial to be informed about the absence or presence of nodal metastasis before commencing any therapy. Accurate information about the presence and location of nodal metastases can prove useful in planning the appropriate nodal dissection to address all involved sites; and avoid unnecessary extensive operations with their side effects on quality of life and increasing the risk of lymphedema. While the need for a highly accurate imaging method for assessment of lymph nodes is paramount, such need is not adequately met by currently available diagnostic imaging methods^6–8^.

Cross-sectional imaging modalities rely on size and morphologic criteria which have limited sensitivity and specificity and thus lack the desired accuracy for characterizing lymph nodes in HNSCC. Identification of metastatic lymph nodes with magnetic resonance imaging (MRI) based on nodal size is limited as demonstrated by variable sensitivity and specificity reported depending on the size criteria used. The limitations of this size-based characterization system are well known: metastases can be present in non-enlarged lymph nodes and not all enlarged nodes are malignant^9^. Identification of lymph node necrosis on conventional imaging is a reliable sign for nodal metastasis, but demonstrates a limited negative predictive value, particularly with smaller nodes. Thus, detection of lymph node metastasis based on nodal size and the presence of necrosis remains difficult.

Currently, advanced functional MRI methods, such as diffusion^10–12^ and perfusion^13^ imaging methods, have been used to detect metastatic lymph nodes. Metastasis in the lymph nodes may be associated with increased cell density leading to alterations in water diffusivity which can be measured as apparent diffusion coefficient (ADC) using diffusion weighted MRI (DWI). Sumi et al.^10^ reported that the metastatic lymph nodes could have a wide range of ADC in HNC and interestingly, the ADC values in the nodes with metastases from poorly differentiated HNC was significantly lower than that from moderately to highly differentiated HNC. However, the accuracy of ADC measurements may be limited by relatively large voxel size and low signal-to-noise ratio (SNR)^10,12^. Dynamic contrast enhanced (DCE)-MRI has been widely used to assess tumor vascularity. A meta-analysis of 43 papers on application of DCE-MRI for lymph node assessment found that the overall accuracy of gadolinium-based DCE-MRI for the detection of nodal metastases is moderate (72% sensitivity and 87% specificity)^13^.

In contrast, positron emission tomography (PET) imaging using 18F-fluorodeoxyglucose (^18^F-FDG) has been found to have high sensitivity (92–100%) in detecting nodal metastases with mixed specificity (77–93%)^14,15^. Investigators in previous studies have evaluated the role of FDG-PET for the detection of lymph node metastasis in patients with HNC mainly by comparing imaging findings with surgical or clinical follow-up findings. Histopathologic correlation has been available in only a few studies^16^. Previous studies investigating fusion techniques of PET and MR imaging found that using these two modalities could improve both sensitivity and specificity for detecting metastatic lymph nodes in HNC^17,18^. However, there is a limited number of studies using the hybrid PET/MRI scanners for assessment of lymph nodes in HNSCC, combined with quantitative data analysis methods for individual modalities.

Therefore, the purpose of this study is to investigate the feasibility of using dynamic FDG-PET with DCE-MRI and DWI in synergy to assess the metastatic status of lymph nodes in HNSCC patients by obtaining quantitative DCE-MRI and FDG-PET kinetic parameters. In this study, we take advantage of using a PET/MR scanner to acquire DCE-MRI, DWI, and dynamic FDG-PET data simultaneously. The hypothesis of this study is that quantitative parameters from all three imaging methods can accurately classify metastatic lymph nodes from normal ones.

## Methods

### Participants

Patients with biopsy-proven head and neck squamous cell carcinoma (HNSCC) (n = 20, mean age 62 ± 16 years ; 15 males and 5 females; Table 1) who were scheduled for cervical lymph node dissection as part of their standard treatment at NYU Langone Medical Center or Bellevue hospital were recruited for this HIPAA-compliant institutional review board-approved study. This research was performed in accordance with relevant guidelines and regulations and written informed consent was obtained from each subject. The primary cancers were squamous cell carcinomas of the oral cavity (n = 14), oropharynx (n = 2), nose (n = 1), larynx (n = 1), and unknown primary (n = 2). Each patient had one research scan using a whole body 3T PET-MR scanner (Biograph mMR, Siemens Healthcare) at the Center for Biomedical Imaging, Department of Radiology, New York University Langone Medical Center, within one week prior to their planned surgery.

**Table 1:** Summary of n = 20 patients’ age, gender, and tumor type, primary location, and stage.

Patient	Age	Gender	Tumor primary location	Stage
1	50	M	Mandible	T2N2aM0
2	80	M	Base of tongue	T1N0M0
3	70	M	Base of tongue	T1N1M0
4	80	M	Nose	T0N2cM0
5	70	M	Tongue	T1N0M0
6	50	M	Buccal mucosa	T1N0M0
7	35	M	Tongue	T2N2bM0
8	93	M	Mandible	T2N0M0
9	65	M	Tongue	T1N0M0
10	73	F	Tongue	T2N2cM0
11	45	M	Tongue	T1N2M0
12	55	M	Floor of mouth	T1N0M0
13	64	F	Gingiva	T4N1M0
14	27	F	Tongue	T1N0M0
15	72	F	Floor of mouth	T1N0M0
16	62	M	Unknown primary	TxN2aM0
17	60	M	Mandible	T4aN2bM0
18	73	F	Mandible	T2N2bM0
19	52	M	Larynx	T4aN1M0
20	57	M	Unknown primary	TxN2bM0

All patients had biopsy-proven HNSCC.

### Identification of nodes

During the surgery, the resected lymph nodes were labeled to identify their nodal levels and served as a guide to locate the corresponding nodes on images^19^. In summary, level I nodes include submental and submandibular nodes, level II nodes are the upper jugular nodes, level III nodes are the middle jugular nodes, level IV nodes are the lower jugular nodes, level V nodes are the posterior triangle nodes, and level VI nodes are the anterior compartment and pretracheal nodes. Surgical specimens were labelled for each nodal level by the operating surgeon and sent for clinical pathology evaluation. Then, on post-contrast MRI, an experienced neuroradiologist (MH with 12 years of experience) manually selected 3D regions of interest (ROI) for internal carotid artery, normal/negative (n = 38) and metastatic/positive (n = 11) nodes that were included in the fields of view of three imaging modalities (DCE-MRI, DWI, and PET) and also specified by the operative note from the neck dissection and pathology reports. The ROIs were drawn so the central necrotic region was included and in every slice where the node was visible. The number of slices per ROI varies from 6 to 25 slices for metastatic nodes and 2 to 25 slices for normal nodes.

### PET data acquisition

Subjects were instructed to fast for at least 6 h before their scans. The PET scan started 1 min before injection of 10 mCi ^18^F-FDG into an antecubital vein and was acquired dynamically for 60 min (38 timeframes; 5 frames of 10 s, 20 s, 30 s, 60 s, and 100 s each, 12 frames of 200 s, and 1 frame of 40 s). Attenuation correction maps were estimated using water and fat images generated from T1-weighted gradient-echo images. PET images were reconstructed using 3D ordinary Poisson ordered subset expectation maximization (OP-OSEM) (127 axial slices, 344 × 344 matrix; 2 × 2 × 2 mm^3^ voxel dimensions)^20^. A post-reconstruction smoothing with a Gaussian filter and kernel width of 2 mm full width at half maximum was applied.

### MRI data acquisition

MRI data were acquired simultaneously during the PET data acquisition. The research imaging sequences described below took a total of 20 min.

DWI was conducted using a twice-refocused spin echo sequence with echo planar readout (TR = 6000 ms, TE 64 ms, flip angle (FA) 180°, matrix 128 × 128 × 27, resolution 2 × 2 × 4.4 mm^3^) with four b-values: 0, 200, 500, and 800 s/mm^2^. The data acquisition with a non-zero b-value was repeated three times with three orthogonal diffusion weighting directions.

T1 mapping was conducted with either MP2RAGE sequence^21^ for five subjects and the variable flip angle (VFA) method^22^ for ten subjects. T1 maps were not available for the remaining five patients, so the average value from 15 subjects was used for the data analysis. MP2RAGE is a variation of standard 3D magnetization-prepared rapid gradient-echo (MPRAGE) sequence, with two inversion times (700 and 2500 ms) with FA of 4° and 5° respectively (TR 5000 ms, TE 2.88 ms, 176 sagittal slices, matrix 256 × 240, resolution 1 × 1 × 1.2 mm^3^). The VFA method used five flip angles using 3D fat suppressed T1-weighted volumetric interpolated breath-hold examination (VIBE) sequence (flip angles = 2°, 4°, 6°, 10°, 15°, TR 10 ms, TE 1.6 ms, 128 axial slices, matrix 256 × 256; resolution 1 × 1 × 2 mm^3^).

DCE-MRI scans were acquired using a golden-angle radial 3D gradient echo sequence (TR = 4.38 ms, TE 2.11 ms, FA 9°, matrix 256 × 256 × 128, resolution 1 × 1 × 2 mm^3^). The baseline images were acquired for 1 min before contrast injection, followed by injection of gadobutrol (Gadavist, Bayer Healthcare Pharmaceuticals) with a concentration of 0.1 mM/kg body weight at the rate of 1 mL/s into the same antecubital vein used for the ^18^F-FDG injection and a saline flush. The scan continued for another 7 min. The dynamic images (76 timeframes, temporal resolution of 5.5 s) were reconstructed using the golden angle radial spare parallel (GRASP) method^23^.

### Image registration

Images from the neck are subject to voluntary and involuntary motion such as swallowing and breathing. In order to minimize such motion artifact, image coregistration across and within modalities was conducted for each subject. In DCE-MRI, the 3D image in the middle timeframe of the scan was empirically selected as the reference image to which individual 3D images of other timeframes were co-registered individually. Co-registration was performed using SimpleElastix software (https//simpleelastix.github.io). We used a multi-resolution registration (2 resolutions) approach with b-spline interpolation (1st order in each resolution, 3rd order in final deformation), advanced Mattes mutual information similarity metric (32 histogram bins), advanced stochastic gradient descent optimizer (4000 max iterations), and b-spline transform (10 mm minimum grid spacing)^24,25^. T_1_ maps were registered to the reference DCE-MRI frame using SimpleITK software^26–28^ with a rigid transform model to match image matrix, voxel dimensions, and orientation. DWI images were first processed using FSL’s TopUp tool to correct susceptibility induced distortions^29,30^ and the Eddy correction tool^31^. After that, DWI volumes were registered to the reference DCE-MRI frame using SimpleITK with mutual information similarity metric, regular step gradient descent optimization, and b-spline transform and interpolation. PET images were resampled to match the DCE-MRI reference frame using FireVoxel (https://wp.nyu.edu/firevoxel).

### Data analysis

After image registration, the dynamic PET, DCE-MRI and DWI data were analyzed using FireVoxel to estimate quantitative parameters related to glucose metabolic rate, vascularity, and cellularity of the selected ROIs.

For DCE-MRI, contrast kinetic model analysis was conducted with the population-based arterial input function (AIF) model proposed by Parker et al.^32^. This was done in view of well-known limitations of individual image-based AIFs^33^. The AIF model was assumed to be the contrast concentration in blood, C_b_(t). C_b_(t) was shifted to match the arrival time of the signal enhancement measured in one of the internal carotid arteries by matching the population AIF peak to the internal carotid artery averaged time activity curve peak. C_b_(t) was converted to the contrast concentration in plasma, C_p_ = C_b_/(1-Hct), where Hct is hematocrit assumed to be 0.42^34^. The mean signal intensity data of a node, S(t), was converted to concentration values C_t_(t) using a linear conversion^35^,

1 $$C_{t} \left( t \right) = \frac{1}{{r_{1} T_{1} }}\left( {\frac{S\left( t \right)}{{S\left( 0 \right)}} - 1} \right)$$

where S(0) is the baseline signal, and r_1_ is the contrast agent relaxivity that was assumed to be 4.5 mM^−1^ s^−1^ here. The linear conversion was used to minimize the uncertainty that could be influenced by the five cases with an assumed T1 value^36^. The two-compartment exchange model (2CXM) was used to estimate mean plasma volume fraction (v_p_), interstitial volume fraction (v_e_), permeability-surface area product (PS), and plasma flow (F_p_)^37^. Volume transfer constant (K^trans^) was then calculated from the estimated F_p_ and PS values; K^trans^ = F_p_(1 − exp(− PS/F_p_)).

The DWI data were used to estimate ADC parametric maps by a monoexponential fit to the signal intensities: ln(S) = ln(S_0_) − b*ADC, where S is the voxel signal, b is the b-value, and S_0_ is the corresponding voxel signal without vascular contribution at b = 0. DWI data with b = 0 was used for the image registration, but not included in the calculation of ADC such that only the data with b ≥ 200 s/mm^2^ were used to minimize the intravoxel incoherent motion effect. We calculated the mean ADC of each lymph node ROI from the estimated ADC maps. DWI field-of-view does not cover every lymph nodes observed in other imaging modalities. Since this paper is investigating assessment of lymph nodes using a combination of DCE-MRI, DWI, and PET parameters, lymph nodes not in DWI field-of-view were excluded because these nodes would be missing diffusion parameter.

The dynamic PET data were analyzed using the irreversible two-compartment model with three parameters; ^18^F-FDG transport rate constant from blood to tissue (K_1_), transport rate constant from tissue to blood (k_2_), and phosphorylation rate constant (k_3_)^38^. This model was the basis of the Sokoloff method used for the estimation of metabolic rate. An image-based AIF was generated for each subject using the previously mentioned neuroradiologist manually created 3D ROIs of the internal carotid artery. A principal component analysis (PCA) method^39^ was used to minimize the partial volume and noise effect in many voxels within the selected ROI for the AIF. As for the input to the PCA analysis, 100 voxels from the ROI with the greatest initial area under the curve (IAUC) for the first 3 min of its dynamic curve were selected. Then, AIF was estimated using the major principal components that make up equal to or more than 95% of the total variance. FireVoxel, our inhouse software was used for parameter estimation of mean K_1_, k_2_, k_3_ and the influx constant K_i_ (K_i_ = K_1_k_3_/(k_2_ + k_3_) related to the metabolic rate.

### Statistical data analysis

We measured mean values of the parameters in each ROI. Once quantitative parameters were estimated, comparisons between normal and metastatic lymph nodes, in terms of DCE-MRI, PET, and DWI parameters, were performed by comparing the median values of the mean ROI parameters between the groups, mainly due to a small number of samples in each group, using the Mann–Whitney U test. This test was computed using SciPy, a free and open-source Python library (https://scipy.org/scipylib). The two-sided 5% significance level was used on all statistical tests. Parameter values reported below are median values with the inter-quartile ranges (IQR) in parentheses unless specified otherwise.

Using logistic regression model from Scikit-learn (C = 1e5, penalty = L2, solver = liblinear), a machine learning library in Python (https://scikit-learn.org/stable/index.html), the PET, DCE-MRI, and DWI parameters along with lymph node volume were fitted to the model individually. The predicted output accuracy was used to rank the parameter or parameters by which yield the highest accuracy. The logistic function was defined as $p\left( x \right) = \frac{1}{{1 + e^{ - f\left( x \right)} }}$, where *f*(*x*) is the linear function, $f\left( x \right) = b_{0} + b_{1} x + \cdots + b_{r} x_{r}$ with variables $b_{0} , b_{1} , \ldots , b_{r}$ as coefficients (predicted weights) and $x, x_{1} , \ldots ,x_{r}$ as parameters, The threshold value for the logistic function output was selected by finding the maximum accuracy using the fitted model.

As an exploratory study, data were classified using two steps; an initial step to use a single parameter with a threshold for 100% specificity to exclude as many normal nodes as possible, followed by a second using a multivariate logistic model of 2 parameters to correctly classify the remaining normal nodes from metastatic nodes.

Receiver operating characteristic (ROC) analysis was used to assess the diagnostic characteristics of three individual parameters and three parameter pairs that best separate normal and metastatic nodes. This analysis was used to show the performance measurement of the logistic regression model at varying thresholds. The ROC curves illustrate trade-off between sensitivity and specificity at different thresholds by plotting sensitivity/true positive rate (TPR) against false positive rate (FPR).

## Results

**Figure 1:**
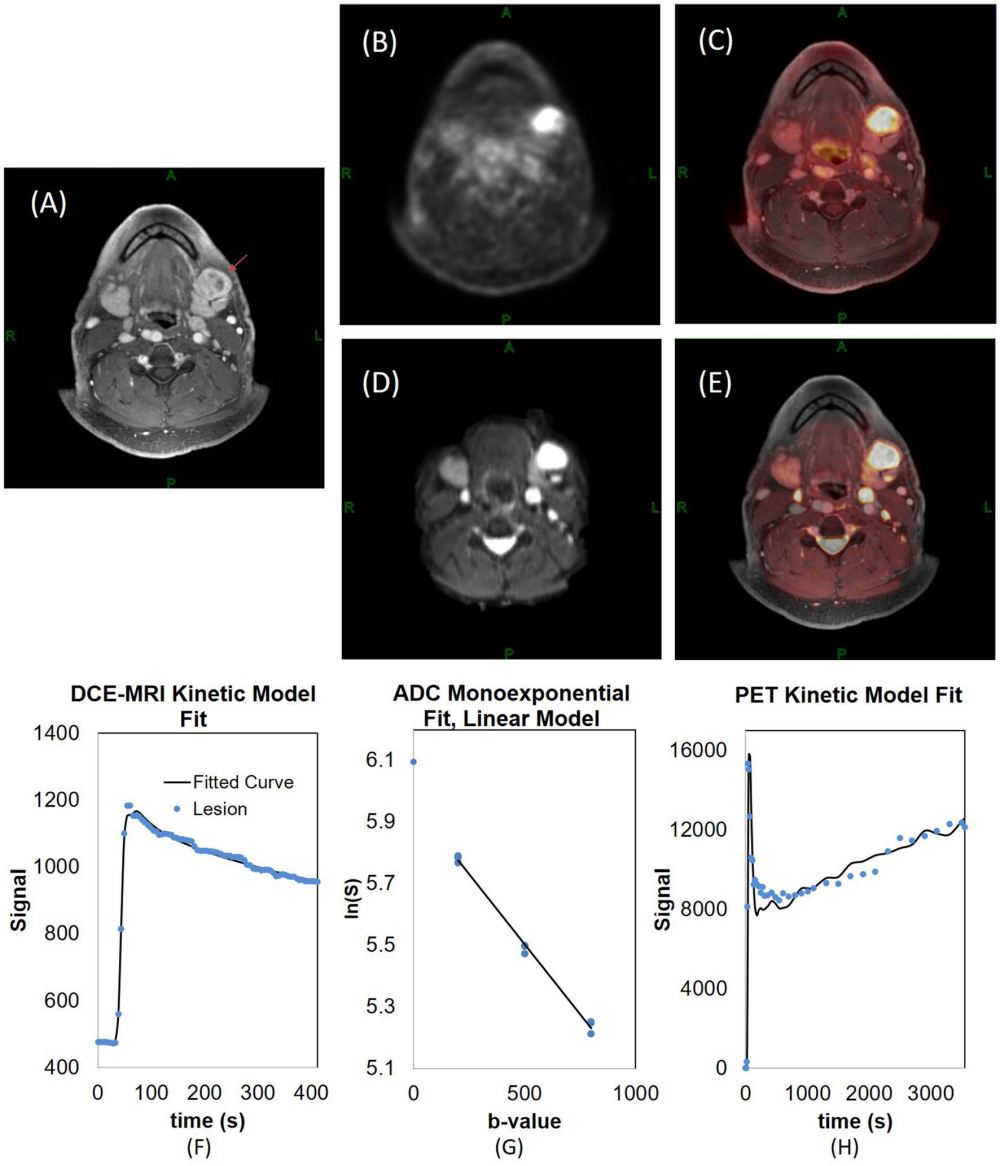
An example of a 50-year-old male patient with HNSCC in the floor of the mouth. A left level-1 metastatic lymph node can be detected in co-registered DCE-MRI (SimpleElastix v0.10.0; https://github.com/SuperElastix/SimpleElastix) (**A**), co-registered 18F-FDG-PET image (**B**), PET activity map overlaid on DCE-MRI (**C**), co-registered DWI b0 image (**D**) and DWI b0 image overlaid on DCE-MRI (**E**). The average data (blue dots) of the left level 1 metastatic lymph node (arrows) are shown for DCE-MRI (**F**), DWI (**G**), and PET (**H**) with their corresponding model fits (solid black lines).

There were 20 HNSCC patients in this study (Table 1). On average, each patient had 3–4 levels dissected, such that there were 79 levels included in this study. All positive nodes were included for the analysis, along with one representative normal node per level. Excluding the nodes not covered in the diffusion imaging protocol with a limited number of slices, a total of 49 nodes (38 normal nodes and 11 metastatic nodes) were included in the quantitative analyses. Figure 1 shows a large metastatic lymph node that can be observed in post-contrast DCE-MR, FDG-PET, and DW images. It demonstrates simultaneous visualization of different measures in the same location (Fig. 1C, E) after the image co-registration and using the same ROI for data analysis of the three modalities. Figure 1 also shows the mean data from a metastatic lesion ROI for three modalities, which demonstrates that the signal models used in these modalities are appropriate. The contrast kinetic model fit to the DCE-MRI data was excellent as shown in Fig. 1F for both the fast wash-in and wash-out phases (F_p_ = 1.49 × 10^−1^ min^−1^, PS = 2.64 × 10^−2^ min^−1^, K^trans^ = 2.42 × 10^−2^ min^−1^, v_p_ = 0.06, and v_e_ = 0.39). In DWI data’s monoexponential fit (Fig. 1G), the log signal decreases linearly within the range of b-values used in this study (ADC = 0.92 µm^2^/ms). The PET data (Fig. 1H) shows an initial vascular phase with a sharp peak followed by a slow irreversible uptake of tracer (K_1_ = 1.43 mL/cm^3^/min, k_2_ = 1.68 min^−1^, k_3_ = 2.16 × 10^−2^ min^−1^, and K_i_ = 1.81 × 10^−2^ mL/cm^3^/min).

### Nodal size and SUV

**Figure 2:**
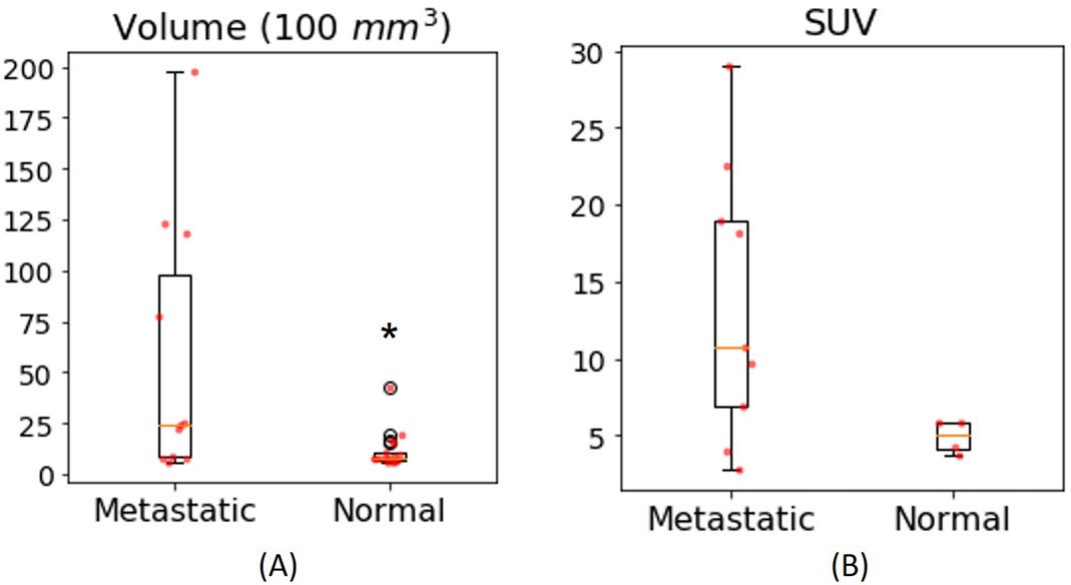
Box–Whisker plots of metastatic and normal lymph node volume in 100 mm^3^ (**A**) and SUV (FDG-avid nodes only) (**B**). Red dots are for individual nodes. *Represents a statistical significance where *p* < 0.05 from Mann–Whitney U test.

Conventional clinical measures, nodal size and specific uptake value (SUV), are shown in Fig. 2. Node size was measured by volume (mm^3^) of ROIs drawn in DCE-MRI. 34% of normal nodes demonstrated volumes greater or equal to the smallest metastatic node. In addition, FDG-avid nodes, considered suspicious for metastasis in clinical reports, were plotted in Fig. 2B. Among the suspicious nodes, four of them were found non-cancerous in pathological evaluation and shown as normal nodes in the plot. Note that the four normal nodes have higher SUV (5.05, IQR = 2.08) than the minimum SUV (2.8) of the metastatic nodes. This shows the shortcomings of only using these clinical measures to determine the metastatic status of suspicious lymph nodes.

### Quantitative MRI measures

**Figure 3:**
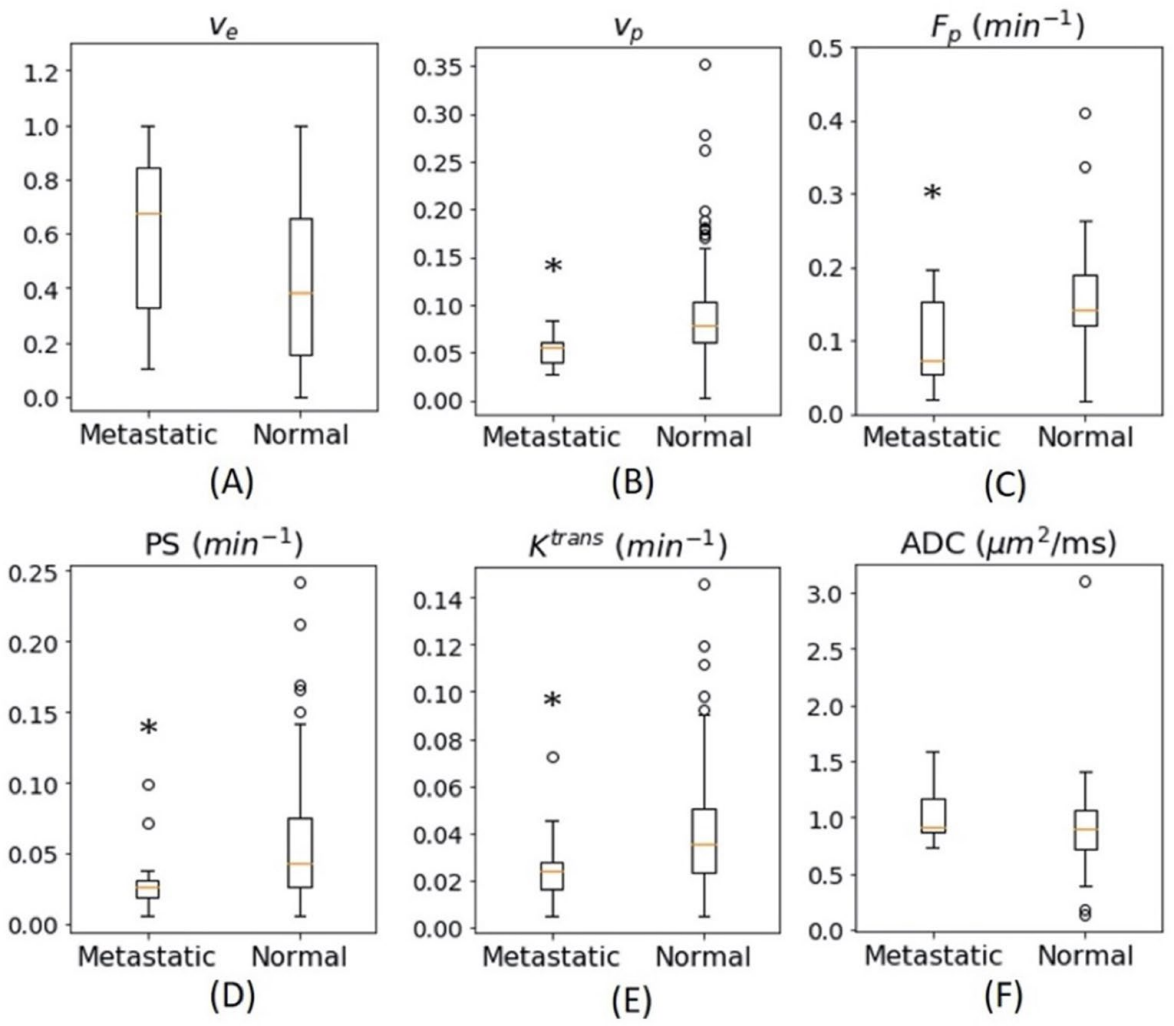
Comparison of DCE-MRI and DWI data between metastatic and normal nodes. Box–Whisker plots show the median and inter-quartile range of v_e_, v_p_, F_p_, PS, K^trans^, and ADC values of normal or metastatic nodes. *Represents a statistical significance where *p* < 0.05 from Mann–Whitney U test.

The estimated DCE-MRI and DWI parameters are summarized in Fig. 3 and Table 2. The DCE-MRI contrast kinetic analysis results show the metastatic lymph nodes had significantly lower median v_p_ (median = 0.06 (IQR = 0.03), *p* < 0.01), F_p_ (7.35 × 10^−2^ (1.03 × 10^−1^) min^−1^, *p* = 0.03), PS (2.64 × 10^−2^ (1.54 × 10^−2^) min^−1^, *p* = 0.03), and K^trans^ (2.42 × 10^−2^ (1.50 × 10^−2^) min^−1^, *p* = 0.02) than those of the normal nodes (v_p_ = 0.08 (0.01), F_p_ = 1.42 × 10^−1^ (7.07 × 10^−2^) min^−1^, PS = 4.24 × 10^−2^ (4.93 × 10^−2^) min^−1^, K^trans^ = 3.79 × 10^−2^ (3.98 × 10^−2^) min^−1^). Of the 11 metastatic nodes, compared to median v_p_ and K^trans^ of normal nodes, 10 had lower v_p_ and 8 had lower K^trans^, of which 8 had lower v_p_ and K^trans^. The median v_e_ of the metastatic nodes (0.67 (0.06)) was greater than that of normal nodes (0.39 (0.05)) but did not reach a statistical significance. The pre-contrast T1 values for DCE-MRI data analysis were measured using either MP2RAGE or VFA method. For the cases with MP2RAGE, the median T1 values for normal and metastatic nodes were 1.30 s and 1.44 s, respectively. For the cases with VFA, they were 1.40 s and 1.55 s, which were not significantly different that those from MP2RAGE. There was no significant difference in ADC between the metastatic nodes (0.96 (0.46) µm^2^) and the normal nodes (0.91 (0.36) µm^2^/ms).

**Table 2:** Summary of the median, first quartile (Q1), and third quartile (Q3) of metastatic and normal ROI kinetic parameters.

	Normal			Metastatic		
	Median	Q1	Q3	Median	Q1	Q3
ADC (µm^2^/ms)	0.911	0.717	1.08	0.962	0.839	1.29
v_p_	7.95 × 10^−2^	6.08 × 10^−2^	0.104	5.57 × 10^−2^*	3.77 × 10^−2^	6.41 × 10^−2^
v_e_	0.385	0.154	0.663	0.673	0.295	0.921
PS (min^−1^)	4.24 × 10^−2^	2.65 × 10^−2^	7.58 × 10^−2^	2.64 × 10^−2^*	1.90 × 10^−2^	3.44 × 10^−2^
F_p_ (min^−1^)	0.142	0.120	0.191	7.35 × 10^−2^*	5.24 × 10^−2^	0.156
K^trans^ (min^−1^)	3.79E−02	2.34 × 10^−2^	6.32 × 10^−2^	2.42 × 10^−2^*	1.58 × 10^−2^	3.08 × 10^−2^
K_1_ (mL/cm^3^/min)	0.816	0.517	1.52	1.20	0.657	2.42
k_2_ (min^−1^)	1.32	0.943	2.10	1.50	1.03	2.34
k_3_ (min^−1^)	3.02 × 10^−3^	1.00 × 10^−4^	6.04 × 10^−3^	1.82 × 10^−2^*	1.05 × 10^−2^	2.34 × 10^−2^
K_i_ (mL/cm^3^/min)	1.81 × 10^−3^	8.93 × 10^−5^	3.42 × 10^−3^	1.23 × 10^−2^*	7.10 × 10^−3^	1.82 × 10^−2^

*A statistical significance where *p* < 0.05 from Mann–Whitney test.

### Dynamic PET measures

**Figure 4:**
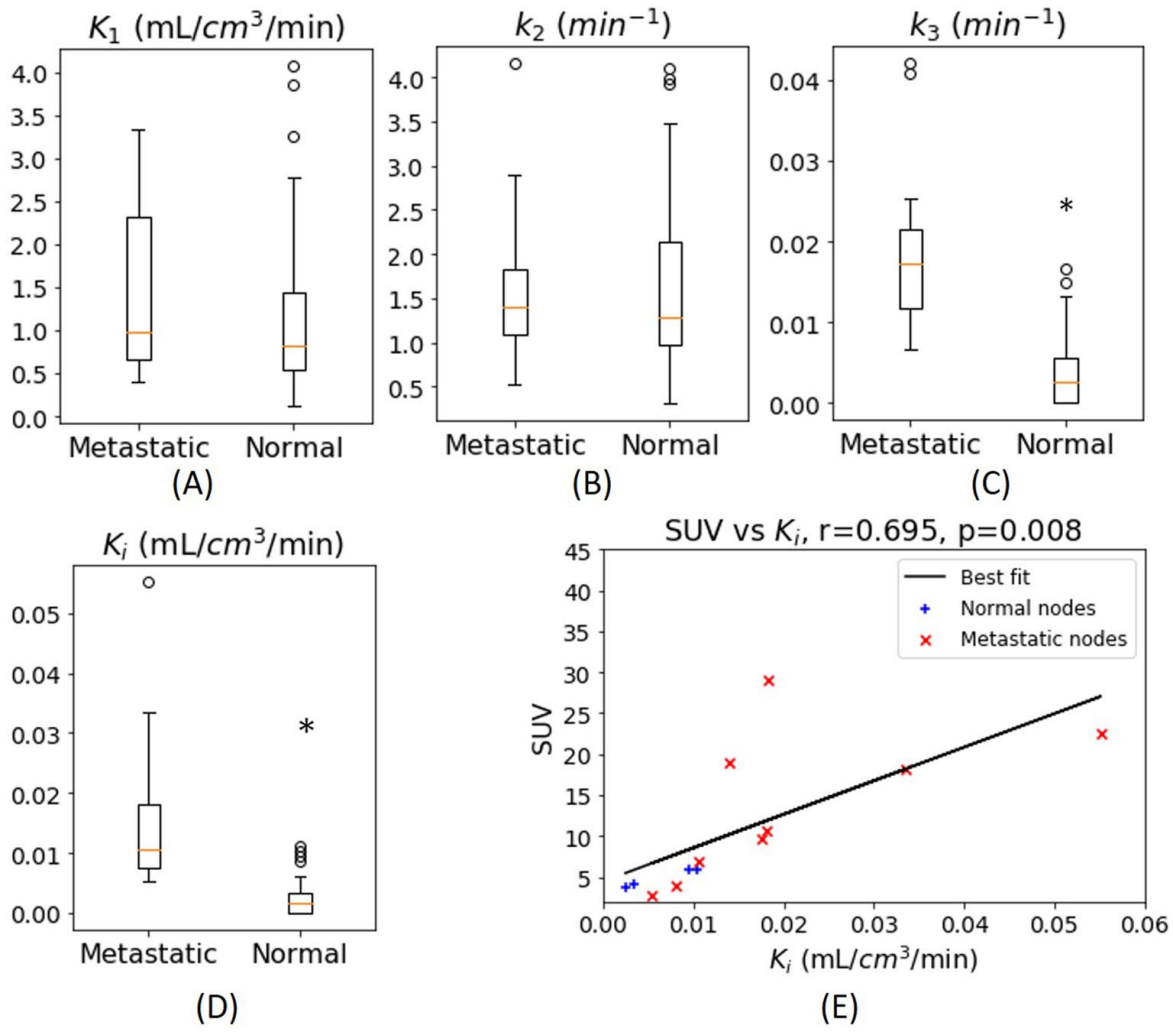
Comparison of dynamic PET data between metastatic and normal nodes. Box–Whisker plots show the median and inter-quartile range of K_1_, k_2_, k_3_, and K_i_ values of normal or metastatic nodes. (E) Scatter plot of K_i_ and SUV in metastatic and normal nodes. Included in the scatter plot are the nodes that were noted as FDG-avid in clinical assessment. These nodes have volumes > 574 mm^3^. *Represents a statistical significance where *p* < 0.05 from Mann–Whitney U test.

Comparisons of the estimated PET pharmacokinetic parameters between metastatic and normal lymph nodes are provided in Fig. 4 and Table 2. Metastatic nodes had significantly higher k_3_ (1.82 × 10^−2^ (1.29 × 10^−2^) min^−1^, *p* = 8.8 × 10^−8^) and K_i_ (1.23 × 10^−2^ (1.11 × 10^−2^) mL/cm^3^/min, *p* = 5.3 × 10^−8^) compared to those of normal nodes (k_3_ = 3.02 × 10^−3^ (5.57 × 10^−3^) min^−1^, K_i_ = 1.81 × 10^−3^ 3.33 × 10^−1^) 5.57 × 10^−3^ mL/cm^3^/min). The K_1_ (1.20 (1.77) mL/cm^3^/min) and k_2_ (1.5 (1.31) min^−1^) of the metastatic nodes were also higher in metastatic nodes than they were in normal nodes (K_1_ = 0.82 (1.00) mL/cm^3^/min, k_2_ = 1.32 (1.15) min^−1^), although there was no statistical significance.

Figure 4E shows a comparison between quantitative parameter, K_i_, and semi-quantitative parameter, SUV, of FDG-avid and enlarged nodes that were considered suspicious for metastasis in clinical PET evaluation. There is a strong correlation (r = 0.70, *p* = 0.008) between K_i_ and SUV.

### Classification of metastatic nodes

**Figure 5:**
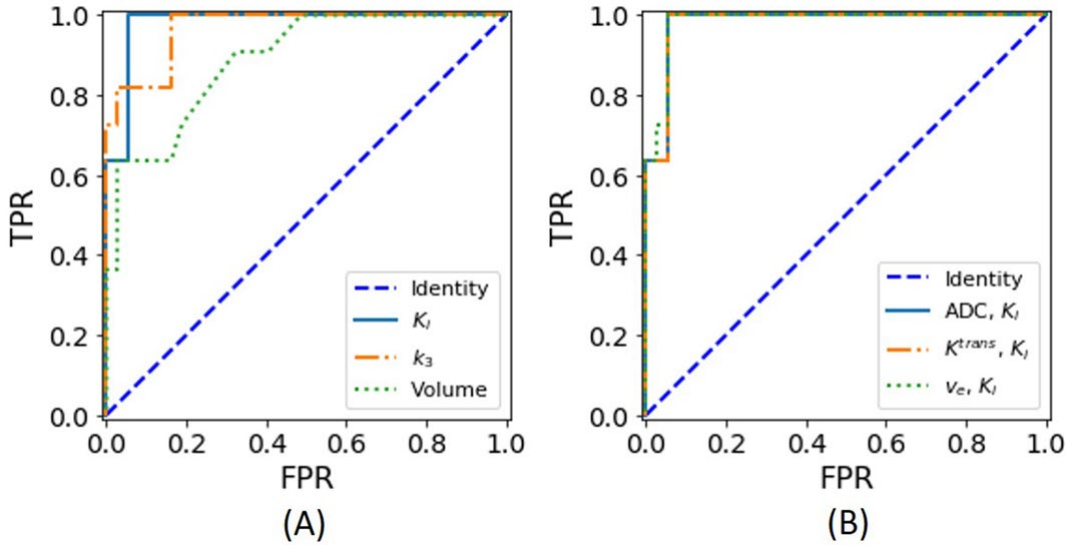
(**A**) ROC curves in single and (**B**) two parameter logistic regression using all 49 lymph nodes.

We investigated whether quantitative PET and MRI measures, individually or combined, can be used for classification of metastatic lymph nodes from normal ones. Using a single parameter model, K_i_ had the highest accuracy of 96%, followed by k_3_ for 94% and volume for 90% (Table 3). The ROC curves of these three parameters are shown in Fig. 5A. There were 10 different pairs of parameters for a logistic regression model that can achieve an accuracy of 96% or higher (Table 3). All 10 pairs included at least one PET parameter. Representative ROC curves of three pairs are shown in Fig. 5B.

**Table 3:** Summary of parameters yielding the most accurate logistic regression prediction from all lymph nodes that overlap in DWI, DCE-MRI, and PET image field-of-view.

Parameters	Threshold	Accuracy	Sensitivity	Specificity	AUC
K_i_	0.18	0.96	1	0.95	0.98
k_3_	0.43	0.94	0.82	0.97	0.97
Volume	0.32	0.90	0.64	0.97	0.89
k_3,_ k_1_	0.45	0.98	0.91	1	0.99
K_3,_ k_2_	0.36	0.98	0.91	1	0.98
ADC, K_i_	0.18	0.96	1	0.95	0.98
K^trans^, K_i_	0.18	0.96	1	0.95	0.98
v_e_, K_i_	0.13	0.96	1	0.95	0.98
v_e_, k_3_	0.15	0.96	1	0.95	0.99
F_p_, K_i_	0.18	0.96	1	0.95	0.98
K_i_, k_3_	0.68	0.96	0.82	1	0.98
K_i_, k_2_	0.18	0.96	1	0.95	0.99
k_3_, Volume	0.54	0.96	0.82	1	0.96

The table includes the threshold used to achieve the highest accuracy.

**Figure 6:**
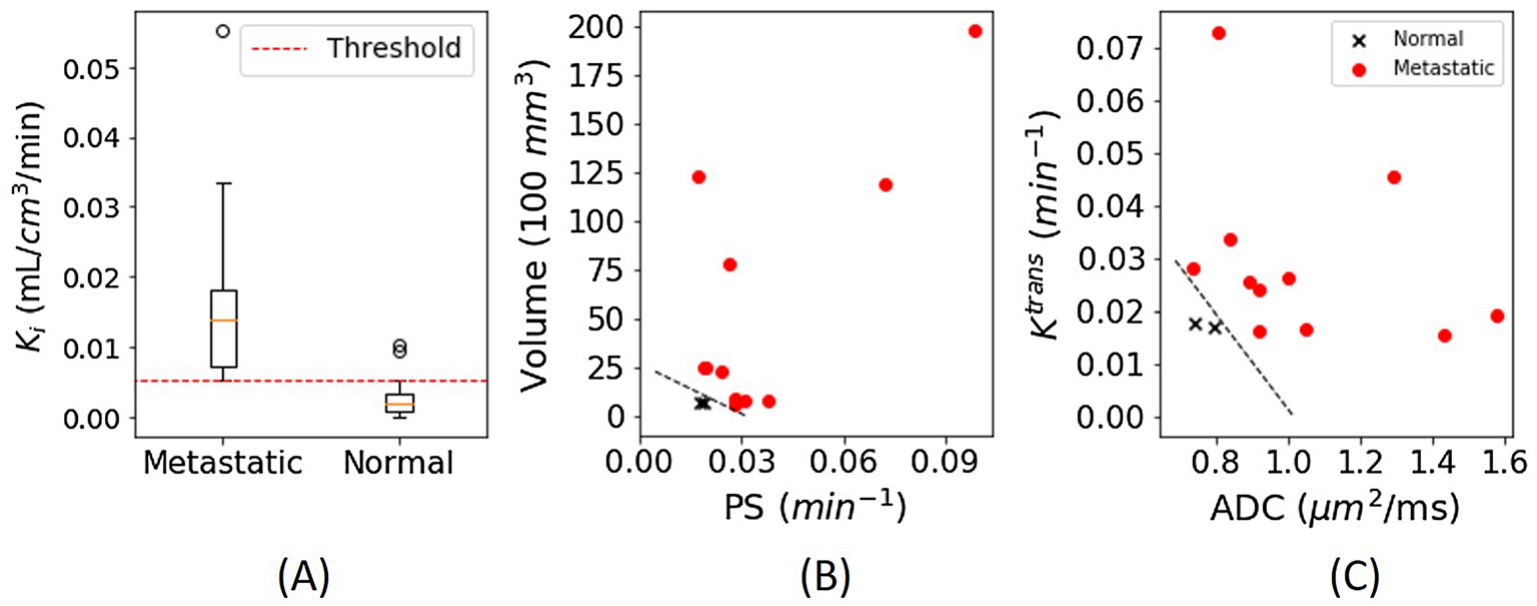
(**A**) Boxplot of K_i_ values of lymph nodes categorized by metastatic and normal nodes showing 5.3 × 10^−3^ mL/cm_3_/min threshold separating metastatic from normal nodes. (**B**) Scatter plot of PS vs volume and (**C**) ADC vs K^trans^ after removing lymph nodes under the 5.3 × 10^−3^ mL/cm^3^/min K_i_ threshold with boundary separating the remaining two normal lymph nodes from metastatic lymph nodes.

We also investigated a two-step approach to improve the accuracy further. As described in the statistical data analysis, the first step was to use a single parameter to exclude as many normal nodes as possible. The second step was to generate a logistic regression model to classify metastatic lymph nodes from the remaining nodes. The analysis found that the best first step was to use K_i_ with cutoff value of 5.3 × 10^−3^ mL/cm^3^/min (Fig. 6A) to remove 36 normal nodes. After this, there were only two normal nodes remaining in the pool with all metastatic nodes. There were 12 pairs of imaging parameters that were able to classify two remaining normal nodes successfully (Table 4). This second step is illustrated by two examples in Fig. 6B, C. Of the 12 two parameter combinations, volume, ADC, and F_p_ were the three most common parameters included and 75% of the 12 combinations included DCE-MRI parameters.

**Table 4:** Pairs of parameters for logistic regression models with prediction accuracy of 100% in classifying the nodes with K_i_ > 5.3 × 10^−3^ mL/cm^3^/min which was the threshold value with 100% negative predictive value to determine normal nodes.

Parameters *x*_1_, *x*_2_	*b*_1_	*b*_2_	Intercept
ADC, PS	28.547	315.060	− 29.648
ADC, K^trans^	28.812	319.573	− 29.314
ADC, F_p_	70.305	− 319.704	− 4.216
ADC, Volume	119.802	16.013	− 208.298
PS, F_p_	384.887	− 49.744	− 0.103
PS, Volume	346.428	0.406	− 10.796
K^trans^, F_p_	392.030	− 51.756	0.687
K^trans^, Volume	352.125	0.440	− 10.587
v_p_, Volume	194.826	0.727	− 16.023
F_p_, k_2_	− 274.030	41.038	− 10.704
k_1_, Volume	18.254	26.594	− 209.330
k_2_, Volume	14.005	10.635	− 95.187

The table includes the model coefficients and intercept.

### FDG-avid non-cancerous nodes

**Figure 7:**
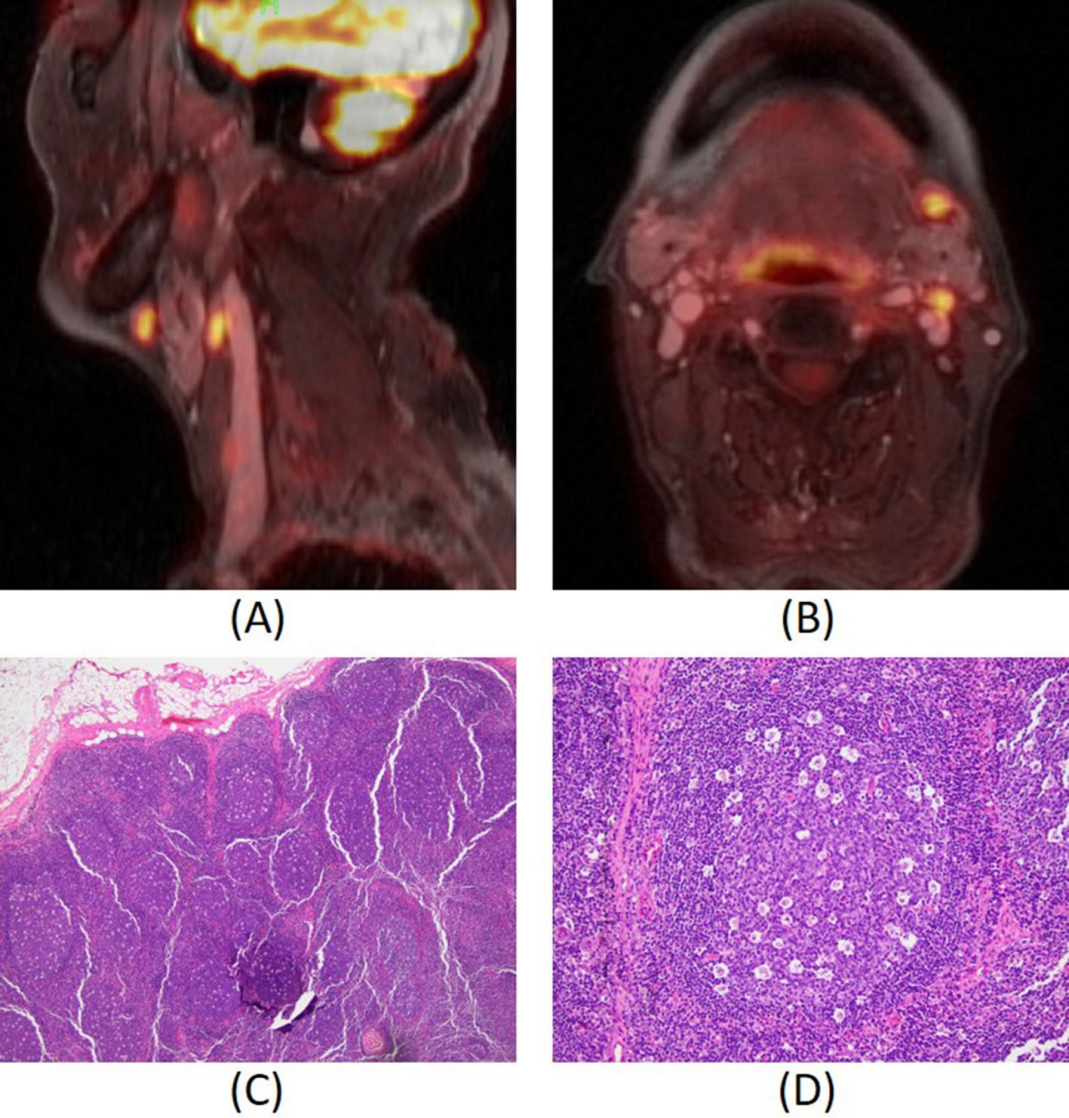
93-year-old male with left mandible SCC PET activity map overlaid on co-registered DCE-MRI (SimpleElastix v0.10.0; https://github.com/SuperElastix/SimpleElastix) (top). Sagittal (**A**) and axial (**B**) slices show 2 regions with SUV = 5.9 and mean K_i_ = 0.01 mL/cm^3^/min, discordant with negative pathology report. Low power view image showing lymphoid follicular hyperplasia (**C**) in lymph node with elevated SUV and high power view image showing tingible body microphages (**D**).

Among the four normal nodes suspected for metastasis (Fig. 4E), two of them had SUV = 5.9 and K_i_ ~ 0.01 mL/cm^3^/min. These two nodes were in level 1 and 2 of one patient as shown in Fig. 7. The subsequent surgical pathology found that these nodes had lymphoid follicular hyperplasia with numerous tingible body macrophages (Fig. 7C, D). The other two normal nodes had SUV of 3.7 and 4.2 without any particular feature to note in pathology.

## Discussion

Quantitative parameters of DCE-MRI, FDG-PET, and DWI may have complementary diagnostic value in detecting metastatic cervical lymph nodes, in addition to using conventional clinical measures, such as volume and SUV. The results from our proof-of-concept study show the feasibility of using multiple parameters such as K_i_, ADC, volume, and F_p_ to improve diagnostic accuracy in identifying metastatic lymph nodes.

### Detection of metastatic lymph nodes using FDG-PET

The results of this study point to significantly higher K_i_ value in metastatic nodes, which indicates abnormally higher metabolism compared to normal nodes. The K_i_ values of the metastatic lymph nodes in our study (0.016 ± 0.014 mL/cm^3^/min) were in good agreement with the values reported from a previous study with HNC patients (0.023 ± 0.004 mL/cm^3^/min)^40^ and another study with a subcutaneous mouse model for non-small cell lung carcinoma (0.024 mL/cm^3^/min)^41^. It was noted that four metastatic nodes in our study had low K_i_ values near the threshold and two normal nodes had K_i_ > 5.3 × 10^−3^ mL/cm^3^/min. This brings up the concern of possible false positives and negatives using FDG-PET biomarkers. Previous FDG-PET studies have reported false positive findings due to inflammation in nodes^14,42^. Schoder et al.^42^ investigated radiographically negative necks (N0) using FDG-PET/CT and noted a relatively high number of false positive nodes probably from high FDG uptake due to low-level lymphadenitis caused by alcohol and tobacco smoking. Similar to our FDG-avid nodes, Schoder et al. found lymphoid follicular and parafollicular hyperplasia in their false positive cases^42^. False positive nodes using PET-CT were also emphasized by Monteil et al.^14^, where they reported 4 of the 5 false positives were inflamed and concluded FDG-PET should be used as a guide in surgery for higher cancer stages, but not a replacement for surgery.

In addition to false positives with difficulty distinguishing inflammation from metastasis, there can also be false negative cases in smaller nodes. Yamazaki et al.^43^ found higher accuracy and less false positives in nodes ≥ 10 mm using FDG-PET compared to CT. True positive nodes had a mean diameter of 13.4 mm while false negative nodes had a mean diameter of 3.1 mm, of which 80% were ≤ 5 mm^43^. Schoder et al. also pointed out that since nodes < 10 mm make up more than half of lymph node metastasis, PET’s low spatial resolution is a limitation^42^. Inaccurate measures in small nodes due to low spatial resolution mentioned in these studies would lead to inaccurate parameter estimations, such as K_i_ estimation. Thus, while FDG-PET shows good separation of metastatic and normal nodes, FDG-PET alone may not be able to differentiate these challenging cases often seen as false positives with inflammation or false negatives of small nodes. The present study results suggest that MRI measures with a higher spatial resolution can be helpful in detecting these small nodes. Further study is warranted to investigate whether there is any inherent difference among imaging modalities in terms of detecting metastatic nodes relatively smaller than their spatial resolutions.

### DCE-MRI for lymph node assessment

Our data shows that metastatic nodes have significantly lower v_p,_ F_p_, PS and K^trans^ than the normal nodes. This observation would not be expected in highly proliferative tumors that actively induces angiogenesis, leading to new vessel formation, and increased permeability and surface area for blood and tissue exchanges. On the other hand, such low level of vascular-related parameters could be explained by necrosis, hypoxia, and elevated interstitial fluid pressure contributing to poor perfusion, commonly observed in aggressive tumors. Previous studies have shown mixed results; significantly lower K^trans^^44^ as well as higher K^trans^^45^ in hypoxic nodes can be observed. Hence, it remains unclear how K^trans^ and other vascular-related parameters can be interpreted in terms of angiogenesis and hypoxia. Imaging hypoxia using fluoromisonidazole (FMISO) could potentially provide useful information in this regard. Further studies are warranted to improve our understanding on how these imaging parameters reflect the tumor physiological status.

The K^trans^ values (0.0026 ± 0.0017 min^−1^) of metastatic and normal nodes observed in our study were relatively lower than those reported in other DCE-MRI studies of HNC that ranged from 0.189 min^−1^ to 0.37 min^−1^^46–49^. This large discrepancy may be from using different AIF measurement methods. Our study used the population-based AIF proposed by Parker et al.^32^ to avoid the influence of the variability in individual AIF measurements from partial volume effect and motion. Since this AIF was based on the measurement in the aorta and iliac arteries, its shape could have higher peak and rising rate than the measurement in a smaller peripheral vessel, resulting in lower K^trans^^50^. Hence, caution should be taken when comparing our DCE-MRI parameters with other studies. However, the comparison of these parameters between metastatic and normal nodes within our study would not be affected by the choice of AIF.

### DWI for lymph node assessment

There are multiple studies that have reported significantly lower ADC in metastatic lymph nodes than that of normal nodes^11,12,51–54^. In the present study, however, we observed that the mean ADC in metastatic nodes was slightly greater than in normal nodes, but no significant difference was found. A similar trend was also observed by Sumi and colleagues that ADC was higher in metastatic nodes than in normal nodes^10^. Interestingly, the studies that reported ADC values lower in metastatic nodes than in normal nodes estimated ADC with low b-values including b = 0^11,12,51–54^. In contrary, our study and the one by Sumi et al.^10^ did not include any b value lower than 200 s/mm^2^ in order to minimize the effect of intravoxel incoherent motion (IVIM) effect and found that the metastatic nodes do not have ADC lower than the normal nodes. Estimation of ADC can be influenced by many factors including IVIM, b-values, signal-to-noise ratio, and how the spatial heterogeneity of the lesions, including necrotic regions, are handled. In order to be able to combine the data from studies from multiple sites, it is imperative to have consensus on the adequate data acquisition and analysis methods specific for cancer imaging^55^. Future studies with such approach would enable us to establish ADC as a robust biomarker to assess the metastatic status of cervical lymph nodes.

### Combining volume, PET, DCE-MRI, and DWI parameters for lymph node assessment

The results of our study suggest the feasibility of reducing the false-positives of FDG-PET and potentially classify lymph nodes with 100% accuracy by supplementing with MRI parameters including the nodal volume measured on MRI. Among individual parameters, K_i_ was the best parameter to discriminate metastatic and normal nodes with 96% accuracy. However, improved accuracy could not be achieved unless a multivariate logistic regression parameter model using volume, PET, and MRI parameters was used as a second step classifier in addition to using K_i_. Future studies with a larger cohort are required to further assess and establish this type of a multivariate diagnostic model to combine PET and MRI parameters for accurate classification of normal and metastatic.

Overall, the present study demonstrates potential value in using FDG-PET and additional MRI quantitative parameters to distinguish metastatic and normal nodes as a proof of concept study. However, there are several limitations to note. Our study took the advantage of a relatively new PET/MR scanner that can acquire both FDG-PET and MRI data simultaneously. It remains to be investigated whether similar results can be achieved using separate PET and MRI scans, despite a bigger challenge of image registration for two separate scans and the time interval between two scans with possibly different physiological conditions. Another limitation was the small cohort of patients included in this study. The result of this study suggests that the additional value of using quantitative MRI can be found with the cases with relatively low FDG uptakes. This was observed with only a handful of cases in this study. Hence, it would be crucial to recruit a larger cohort of patients for future studies. The current study was also limited to assessing the average values of the PET and MRI parameters, rather than the histogram or textual measures. There are many small nodes, particularly normal ones, which are not appropriate for histogram or textual analysis. On the other hand, there are relatively large nodes which could benefit from analyzing the spatial distribution of quantitative parameters. This histogram analysis may distinguish voxels with necrosis from other voxels in the ROI. However, it was beyond the scope of our study to investigate how to assess spatial distributions appropriately when nodes have a wide range of sizes.

## Conclusion

In this study, we assessed the feasibility of using simultaneous PET-MR imaging for assessment of metastatic status of cervical lymph nodes in HNSCC patients prior to surgery. We were able to co-register the multi-modality images and extract quantitative parameters that represent the status of nodes in terms of glucose metabolic rate, perfusion and diffusion. These parameters were successfully used to classify metastatic and normal nodes, albeit a small cohort for this proof-of-concept study. Our results suggest that quantitative MRI parameters provide additional value in distinguishing metastatic nodes, particularly among small nodes, when used together with FDG-PET. Future studies with a large cohort are warranted to further investigate the synergistic role of FDG-PET and MRI for accurate assessment of metastatic lymph nodes in head and neck cancer.
